# Low Level Viremia in Early HIV RNA Kinetics in Naïve People Living With HIV Treated With Bictegravir/Emtricitabine/Tenofovir Alafenamide or Dolutegravir/Abacavir/Lamivudine: An Observational Multicenter Retrospective Study

**DOI:** 10.1002/hsr2.73493

**Published:** 2026-11-10

**Authors:** Nicola Bonadiman, Monica Basso, Maria Cristina Rossi, Mario Giobbia, Gloria Tomarchio, Gaia Doro, Carla Casarin, Pietro Luigi Rocco, Francesca Cattelan, Giuliana Battagin, Saverio G. Parisi

**Affiliations:** ^1^ Department of Molecular Medicine University of Padova Padova Italy; ^2^ Infectious Disease Unit Treviso Hospital Treviso Italy; ^3^ Infectious Diseases Unit, dell'Angelo Hospital Venezia Italy; ^4^ Infectious Diseases Unit, Vicenza Hospital Vicenza Italy

**Keywords:** early kinetics, HIV, integrase inhibitors, low level viremia, naïve patients

## Abstract

- Integrase inhibitors have a pivotal role in antiviral therapy
- A quarter of PLWH had LLV after 3 months of treatment
- PLWH with LLV had immunological parameters lower than PLWH with virological suppression but comparable to those with partial response

## 1 Introduction

Integrase strand transfer inhibitors (InSTI) are recommended as first‐line antiretroviral therapy (ART) for naïve people living with HIV (PLWH). Most PLWH achieve virological suppression, defined as plasma HIV RNA < 50 copies/ml. The two‐drug combinations bictegravir/emtricitabine/tenofovir (BIC/FTC/TAF) and dolutegravir/abacavir/lamivudine (DTG/ABC/3TC) demonstrate comparable efficacy at 48 weeks of treatment [1, 2].

Viral decay after ART initiation is a multistep process, driven initially by the clearance of short‐lived infected cells, followed by the slower loss of longer‐lived and latently infected CD4+ cells, which may contribute to the persistence of low‐level viremia (LLV), together with ongoing replication in lymphoid tissue reservoirs [3, 4].

LLV is defined as the detection of plasma HIV RNA values ranging from 50 to 200 copies/ml, despite adherence to therapy and the absence of drug resistance. Although this phenomenon is not uncommon, its clinical significance remains a topic of debate. It has been speculated that LLV is associated with immune activation and an inflammatory state [5, 6].

The aim of this study is to describe the early (3 months) HIV RNA virological response in naïve PLWH treated with BIC/FTC/TAF or DTG/ABC/3TC, as determined by the treating physician in a real‐life setting. We focus on characteristics of the PLWH who achieved LLV (HIV RNA 50–200 copies/mL) compared to those who attained successful virological response (HIV RNA < 50 copies/mL) or partial virological response (HIV RNA ≥ 201 copies/ml).

## 2 Material and Methods

This retrospective cohort study was conducted in accordance with the STROBE guidelines [7].

Naïve PLWH outpatients who underwent a pretreatment visit from February 2018 to December 2023 were consecutively enrolled in the Infectious Diseases Units of Mestre, Treviso, and Vicenza (Italy). All the PLWH with available virological response data after 3 months of treatment with BIC/FTC/TAF or DTG/ABC/3TC were enrolled in the study.

Response to InSTI‐based ART at the third month (T3) was classified as follows: virological suppression (plasma HIV RNA < 50 copies/mL), low‐level viremia (plasma HIV RNA 50–200 copies/mL), and partial response (plasma HIV RNA ≥ 201 copies/mL).

All data reported in the study were part of the routine monitoring of treated PLWH. The study was conducted in accordance with the Helsinki Declaration and with Good Clinical Practice: the Ethics Committee of Padova Province (protocol code 0033219) approved the study and written informed consent was obtained by the PLWH. Viroimmunological parameters were analyzed in the two cohort of PLWH and in the overall study population.

The main parameters evaluated at T0 (before starting ART) included CD4+ cell count at nadir, the presence of HBV and HCV co‐infection, and median plasma HIV RNA. Parameters evaluated at both T0 and T3 included CD4+ cell count and percentage, as well as the CD4/CD8 ratio.

Continuous variables were described as median and interquartile range, while categorical variables were presented as absolute numbers and percentage.

Mann–Whitney test was applied to test a variable in two independent groups of subjects while the Wilcoxon signed‐rank test was chosen if the observations were paired: both tests were used to analyze continuous variables. Categorical data identified in two independent groups were evaluated with Chi‐square test and Fisher's exact test, based on the expected counts in the table [8]. All *p*‐values were two‐sided. A *p‐*value < 0.05 was considered statistically significant. Statistical analysis was performed using MedCalc Statistical Software version 23.5.2 (MedCalc Software Ltd, Ostend, Belgium; https://www.medcalc.org; 2026).

## 3 Results

One hundred thirty‐one PLWH started ART and 124 PLWH (M/F 86/38) were included in the study; 88 PLWH (71%) were treated with BIC/FTC/TAF, and 36 PLWH (29%) received DTG/ABC/3TC. Seven PLWH were excluded because 5 discontinued ART and 2 were lost to follow‐up. Overall, median age was 42 (IQR 33–52) years, with a median CD4+ cell count at nadir of 160 (IQR 44–405) cells/mm^3^, CD4+ cell count at T0 of 160 (IQR 47–407) cells/mm^3^, CD4+ percentage at T0 of 12.2 (IQR 5.9–20.89) %, a CD4/CD8 ratio of 0.20 (IQR 0.08–0.33), and a median plasma HIV RNA at T0 of 228,500 (57,500–877,500) copies/mL.

No significant difference in baseline characteristics between the BIC/FTC/TAF and DTG/ABC/3TC regimen groups was detected (Table S1).

At T3, a total of 68 (54.8%) PLWH achieved virological suppression, 31 (25%) PLWH experienced LLV, and partial response was observed in 25 (20.2%) PLWH (Figure S1): the relative frequencies of the virological response categories were comparable between the two ART regimens

PLWH with LLV at T3 had a median pre‐treatment CD4+ cell count of 124 cells/mm^3^, a CD4+ percentage of 8%, a CD4/CD8 ratio of 0.10, and a median baseline plasma HIV RNA of 462,000 copies/mL. Compared with PLWH who achieved virological suppression, this group showed significantly lower immunological parameters (CD4+ count, CD4+ percentage, and CD4/CD8 ratio) and markedly higher baseline viremia. These values were instead comparable to those observed in PLWH with a partial virological response. Consistently, individuals who achieved virological suppression had a median baseline CD4+ cell count > 200 cells/mm^3^ and a median baseline plasma HIV RNA < 100,000 copies/mL. A detailed description of the characteristics of the three cohorts is provided in Table 1.

**Table 1:** Pre‐treatment (T0) characteristics of PLWH who achieved a plasma HIV RNA value < 50 copies/ml, of 50–200 copies/mL and ≥ 201 copies/mL after 3 months of antiretroviral therapy (T3). Continuous variables are expressed as median and interquartile range.

	PLWH with plasma HIV RNA < 50 copies/ml at T3 (*n* = 68)	PLWH with plasma HIV RNA 50–200 copies/ml at T3 (*n* = 31)	PLWH with plasma HIV RNA ≥ 201 copies/ml at T3 (*n* = 25)	*p* (HIV RNA < 50 copies/ml vs HIV RNA 50–200 copies/mL)	*p* (HIV RNA < 50 copies/ml vs HIV RNA ≥ 201 copies/mL)	*p* (HIV RNA 50–200 copies/ml vs HIV RNA ≥ 201 copies/mL)
Age at diagnosis (years)	40 (30‐47)	43 (37–54)	45 (37‐53)	**0.03**	0.11	0.86
Male gender, n (%)	45 (66.2)	22 (71)	19 (76)	0.63	0.36	0.67
Italian origin, n (%)	42 (61.8)	17 (54.8)	14 (56)	0.51	0.61	0.93
CD4+ cell count (cells/mm^3^) at nadir	264 (69‐564)	124 (59‐163)	40 (16‐164)	**< 0.001**	**< 0.001**	0.13
CD4+ cell count (cells/mm^3^)at T0	253 (89‐560)	124 (60‐163)	40 (16‐164)	**< 0.001**	**< 0.001**	0.13
CD4+ percentage at T0	19.2 (8.1‐24.5)	8 (5.8‐13.8)	6.6 (4.3‐13.9)	**< 0.001**	**< 0.001**	0.61
CD4/CD8 ratio	0.29 (0.10‐0.43)	0.10 (0.07‐0.20)	0.11 (0.07‐0.21)	**< 0.001**	**0.006**	0.73
Positive HCV serology, I (%)	7 (10.3)	2 (6.5)	2 (8)	0.71	> 0.99	> 0.99
Positive HBsAg, n (%)	4 (5.9)	0	3 (12)	0.3	0.38	0.08
Negative HBsAg and positive HBcAb positive, *n* (%)	14 (21.9)	10 (32.2)	2 (9.1)	0.27	0.22	0.09
Plasma HIV RNA (copies/mL)	85350 (25583‐409500)	462000 (187250‐ 1212500)	1033712 (274500‐2427839)	**< 0.001**	**< 0.001**	0.15

*Note:* Bold values indicate significant *p* values.

Abbreviation: PLWH: people living with HIV.

In all three categories of plasma HIV RNA response at T3, a significant increase in median values of CD4+ cell count and percentage and of CD4/CD8 ratio was observed compared to pre‐treatment values: nevertheless, immunological recovery parameters at T3 were lower in LLV and with PR with respect to PLWH with VS (Table 2).

**Table 2:** Description of the immunological parameters after 3 months of antiviral therapy according to the 3 classes of virological response (panel a) and of the statistical analysis (panel b). Continuous variables are expressed as median and interquartile range.

(a)						
	PLWH with plasma HIV RNA < 50 copies/ml at T3 (*n* = 68)	*p* (vs T0 value)	PLWH with plasma HIV RNA 50–200 copies/ml at T3 (*n* = 31)	*p* (vs T0 value)	PLWH with plasma HIV RNA ≥ 201 copies/ml at T3 (*n* = 25)	*p (*vs T0 value)
CD4+ cell count (cells/mm^3^) at T3	397 (161–633)	**< 0.001**	254 (179–394)	**< 0.001**	199 (116–322)	**< 0.001**
CD4+ cells percentage at T3	23.2 (12.1– 30.3)	**< 0.001**	15.1 (10.1–23.02)	**< 0.001**	15 (7.8–19.7)	**< 0.001**
CD4/CD8 ratio at T3	0.40 (0.19–0.60)	**< 0.001**	0.22 (0.14–0.38)	**< 0.001**	0.22 (0.11–0.37)	**0.002**

(b)			
	*p* (HIV RNA < 50 copies/ml vs HIV RNA 50‐200 copies/ml)	*p* (HIV RNA < 50 copies/ml vs HIV RNA ≥ 201 copies/ml)	*p* (HIV RNA 50‐200 copies/ml vs HIV RNA ≥ 201 copies/ml)
CD4+ cell count (cells/mm^3^) at T3	**.04**	**.004**	.17
CD4+ percentage at T3	**.02**	**.001**	.20
CD4/CD8 ratio at T3	.**02**	**.01**	.69

*Notes:* Bold values indicate significant *p* values.

Abbreviations: PLWH: people living with HIV; T3: third month of antiretroviral therapy.

## 4 Discussion

This study describes the early virological response to InSTI‐based first‐line ART in an unselected cohort of PLWH followed in a real‐life setting. Notably, a quarter of participants exhibited plasma HIV RNA values within the LLV range.

Interestingly, these PLWH had significantly different pre‐treatment median CD4+ counts, percentages, CD4/CD8 ratios, and plasma HIV RNA values compared to those who achieved virological suppression, while their values were comparable to those of PLWH with plasma HIV RNA ≥ 201 copies/mL.

In our study, the frequency of PLWH achieving virological suppression was 54.8%, which is lower than previous studies reporting frequencies > 70%. However, participants in studies by Ugarte et al. [9], Podzamczer et al. [10], and Gallant et al. [2] had median CD4 cell counts > 350 cells/mm^3^ and median plasma HIV RNA value < 100,000 copies/mL. These authors did not report the percentage of PLWH with plasma HIV RNA levels between 50 and 200 copies/mL. However, their higher rates of virological suppression suggest that baseline factors known to be associated with persistent LLV during long‐term ART, such as HIV‐1 RNA levels > 100,000 copies/mL and CD4 + T‐cell counts ≤ 200 cells/μL [11], may also influence the early response to ART. Consistent with this interpretation, PLWH with LLV after 3 months of treatment in our study had a median baseline HIV RNA level of 462,000 copies/mL and a median baseline CD4 + T‐cell count of 124 cells/mm^3^.

A Delphi consensus [12] recommended measuring total HIV DNA levels in PLWH with LLV detected after 6 months of first‐line ART, given the correlation between LLV and slower reservoir decay [13]. Although patients in our study were on ART for a shorter duration, reservoir levels decrease after longer periods of successful therapy [14]. Therefore, we can assume that data from 3 and 6 months are comparable in this context, making early HIV RNA load data valuable when 6‐month LLV data are unavailable and supplementary otherwise.

The primary limitation of this study is its design, which focuses solely on early kinetics without correlating with virological response at further follow‐up points. However, PLWH who achieve virological suppression may have been switched to a two‐drug regimen at the discretion of the treating physician, thus potentially excluding them from longitudinal analysis, which would only include those who do not achieve a complete response and, moreover, LLV detected in two consecutive within 3–9 months after treatment start is linked to virological failure [15].

## 5 Conclusion

In conclusion, a significant percentage of naïve PLWH treated with an integrase‐based regimen exhibited plasma HIV RNA values within the range of LLV after 3 months of treatment. This cohort demonstrated baseline viroimmunological parameters that were significantly different from those of PLWH with a successful response. However, the small sample sizes of the PLWH cohorts with LLV and PR may have limited the statistical power of our analysis, potentially contributing to the absence of statistically significant differences between the two groups.

Further studies in larger cohorts are warranted to confirm these findings and, through longitudinal analyses, to elucidate the clinical implications of low‐level viremia detected 3 months after treatment initiation on subsequent patient outcomes.

## Author Contributions

**Nicola Bonadiman:** investigation, visualization, writing – review and editing; writing – original draft. **Monica Basso:** formal analysis, resources, writing – original draft; writing – review and editing. **Maria Cristina Rossi:** investigation. **Mario Giobbia:** investigation. **Gloria Tomarchio:** visualization. **Gaia Doro:** visualization. **Carla Casarin:** investigation. **Pietro Luigi Rocco:** investigation. **Francesca Cattelan:** investigation. **Giuliana Battagin:** investigation. **Saverio G. Parisi:** conceptualization, project administration, resources, supervision, writing – review and editing, writing – original draft.

## Disclosure

The corresponding author Monica Basso affirms that this manuscript is an honest, accurate, and transparent account of the study being reported; that no important aspects of the study have been omitted; and that any discrepancies from the study as planned (and, if relevant, registered) have been explained.

## Conflicts of Interest

The authors declare no conflicts of interest.

## Supporting information


Supporting File 1

Supporting File 2

## Data Availability

The data presented in this study are available on reasonable request from the corresponding author. The data are not publicly available because they contain information that could compromise the privacy of participants.
