# Supplementary material for: Low Level Viremia in Early HIV RNA Kinetics in Naïve People Living With HIV Treated With Bictegravir/Emtricitabine/Tenofovir Alafenamide or Dolutegravir/Abacavir/Lamivudine: An Observational Multicenter Retrospective Study
Source: Health Sci Rep. 2026 Nov 10;9(11):e73493. doi: 10.1002/hsr2.73493 (PMC13749419; doi:10.1002/hsr2.73493)
Supplement: Supplementary file 1 [file HSR2-9-e73493-s002.pdf]

Figure S1. Distribution of the frequency of the three different possible virological responses at T3. Data are expressed both as absolute value and as relative percentage.

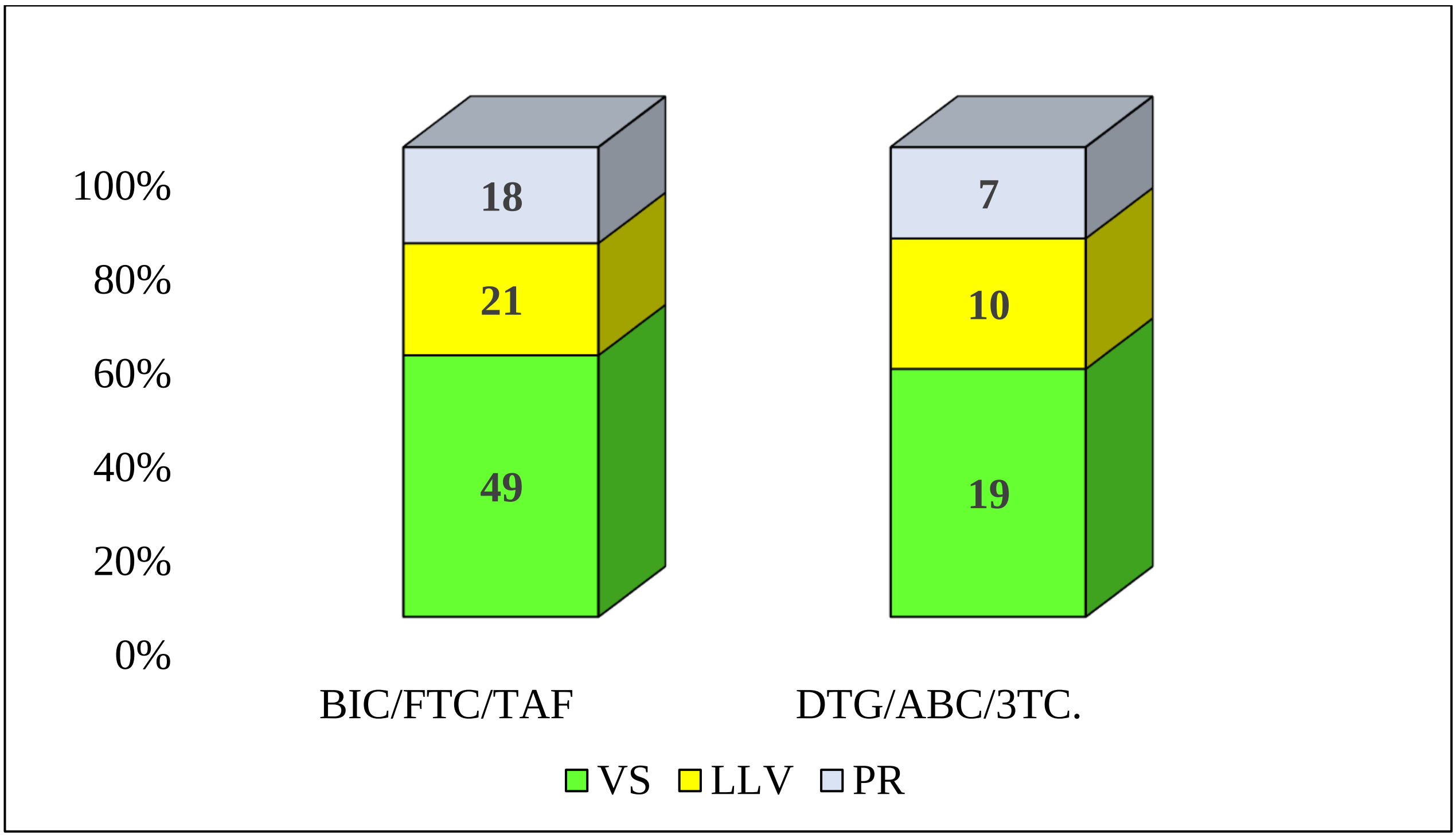

BIC/FTC/TAF: bictegravir/emtricitabine/tenofovir alafenamide

DTG/ABC/3TC: dolutegravir/abacavir/lamivudine

VS: HIV RNA < 50 copies/ml after 3 months of antiretroviral treatment (T3)

LLV: HIV RNA 50–200 copies/ml after 3 months of antiretroviral treatment (T3)

PR: HIV RNA ≥ 201 copies/ml after 3 months of antiretroviral treatment (T3)
