# Supplementary material for: Low Level Viremia in Early HIV RNA Kinetics in Naïve People Living With HIV Treated With Bictegravir/Emtricitabine/Tenofovir Alafenamide or Dolutegravir/Abacavir/Lamivudine: An Observational Multicenter Retrospective Study
Source: Health Sci Rep. 2026 Nov 10;9(11):e73493. doi: 10.1002/hsr2.73493 (PMC13749419; doi:10.1002/hsr2.73493)
Supplement: Supplementary file 2 [file HSR2-9-e73493-s001.docx]

**Table S1.** Study population characteristics at the treatment start (T0) in PLWH treated with BIC/FTC/TAF and treated with DTG/ABC/3TC. Continuous variables are reported as median and interquartile range.

|  | PLWH treated with BIC/FTC/TAF (n=88) | PLWH treated with DTG/ABC/3TC (n=36) | p |
| --- | --- | --- | --- |
| Age at diagnosis (years) | 41 (32.5- 48.5) | 42 (34-54) | 0.45 |
| Male gender, n (%) | 61 (69.3) | 25 (69.4) | 0.98 |
| Italian origin, n (%) | 49 (55.7) | 24 (66.7) | 0.26 |
| CD4+ cell count (cells/mm^3^) at nadir | 160 (40-405) | 160 (52-406) | 0.8 |
| CD4+ cell count (cells/mm^3^) | 160 (40- 447) | 160 (57-385) | 0.88 |
| CD4+ percentage | 12 (6-20) | 13 (4.9-22.1) | 0.82 |
| CD4/CD8 ratio | 0.20 (0.09- 0.35) | 0.11 (0.06-0.31) | 0.26 |
| Positive HCV serology, n (%) | 9 (10.2) | 2 (5.6) | 0.5 |
| Positive HBsAg, n (%) | 5 (5.7)) | 2 (5.6) | 1 |
| Negative HBsAg and positive HBcAb positive, n (%) | 21 (25.3) | 5 (14.7) | 0.32 |
| Plasma HIV RNA (copies/ml) | 278313 (55300-1020201) | 203675 (87600- 821500) | 0.7 |

PLWH: people living with HIV

BIC/FTC/TAF: bictegravir/emtricitabine/tenofovir

DTG/ABC/3TC: dolutegravir/abacavir/lamivudine
